# User Engagement on a Novel Educational Health Intervention Aimed at Increasing HPV Vaccine Uptake in Hong Kong: a Qualitative Study

**DOI:** 10.1007/s13187-022-02183-7

**Published:** 2022-07-06

**Authors:** Janita Pak Chun Chau, Suzanne Hoi Shan Lo, Laveeza Butt, Vivian Wing Yan Lee, Grace Chung Yan Lui, Alexander Yuk Lun Lau

**Affiliations:** 1 The Nethersole School of Nursing, Faculty of Medicine, The Chinese University of Hong Kong, Shatin, N.T. Hong Kong; 2 Centre for Learning Enhancement and Research, The Chinese University of Hong Kong, Shatin, N.T. Hong Kong; 3 Division of Infectious Diseases, Department of Medicine and Therapeutics, Faculty of Medicine, The Chinese University of Hong Kong, Shatin, N.T. Hong Kong; 4 Division of Neurology, Department of Medicine and Therapeutics, Faculty of Medicine, The Chinese University of Hong Kong, Shatin, N.T. Hong Kong

**Keywords:** Adolescents, HPV vaccine, Human papillomavirus, Qualitative, Vaccine hesitancy

## Abstract

HPV vaccine uptake rates are suboptimal in Hong Kong. A multi-disciplinary school-based HPV health-promotion programme (MDL-SHPVP) aimed at raising HPV knowledge levels and increasing vaccine uptake has therefore been developed to address vaccine hesitancy. This qualitative study was conducted to collect user feedback and identify the strengths and limitations of the educational resources developed for the programme among key vaccination stakeholders including adolescent girls and their mothers. Twenty-six participants including eight mother-daughter dyads, four teachers, three social workers, two school principals and one school nurse were recruited. To cater to the diverse audience, ten educational videos, three animations, a digital game and one booklet were developed for the programme and distributed to the participants for viewing. Semi-structured interviews were then conducted to collect feedback on the acceptability and effectiveness of the resources. Interviews were audio-recorded, transcribed verbatim, and resulting data were thematically analysed. Three themes and six sub-themes emerged. The educational materials were well-received and effective in raising HPV-knowledge levels, generating confidence in vaccine safety and effectiveness, and boosting vaccination intention. Some doubts regarding vaccine necessity remained, and recommendations for improving resource presentation and accessibility were provided. Our findings suggest that the MDL-SHPVP has the potential to boost HPV vaccine uptake. Future studies may explore educational interventions which target to increase not only HPV vaccination intention but also the sense of urgency so as to encourage timely vaccination for adolescents at the ideal age. Study findings may also provide directions for the development of future health education interventions.

## Introduction

Human papillomavirus (HPV) infection is the most common sexually transmitted infection in the world. It is also the necessary cause of cervical cancer, the fourth most common cancer among women worldwide and the seventh most common in Hong Kong (HK) [1, 2]. Studies show that existing HPV vaccines are effective in the prevention of cervical cancer as they protect against most cancer-causing HPV subtypes and are endorsed by national health institutions worldwide [3]. As the vaccination shows higher efficacy in persons who have never had an HPV infection, it is recommended for girls aged 9–14 who have yet to become sexually active [1]. However, due to a combination of factors including low awareness, misinformation, distrust, inaccessibility, social attitudes, and more, vaccination uptake worldwide remains suboptimal [4, 5]. In HK as well, though the HPV vaccine has recently been included in the vaccination schedule of the Childhood Immunisation Programme for primary-school aged children [6], there is still a sizeable population of young adolescents who, despite being at the recommended vaccination age, will miss out on the free vaccination. It is therefore imperative to communicate the necessity of HPV vaccination to this particular group of adolescents and their parents in order to encourage them to receive the vaccination at their own initiative.

Health education is a potentially effective solution to encourage vaccine acceptance and combat hesitancy. In a previous study, it was found that misconceptions and low knowledge levels of HPV are common among various stakeholders in Hong Kong [7]. By highlighting the relevance of the vaccine, addressing concerns regarding side effects and more, education can empower people in the decision-making process and instil confidence regarding the value of HPV vaccination. This is demonstrated in a study conducted by Kwan et al. which showed that an educational programme on cervical cancer was able to generate high vaccine acceptability and increase participant intention for vaccination [8]. However, while the study provided valuable evidence regarding the benefits of targeted health education, vaccine uptake was not reported, thus casting doubt on the actual success of such interventions. Further studies which investigate the impact of such interventions in genuinely raising vaccination rates are thus needed.

To address this research gap, we aimed to assess the effectiveness of a novel multi-disciplinary school-based HPV health-promotion programme (MDL-SHPVP) which we developed, in raising knowledge levels regarding HPV, improving perceptions of HPV vaccination, and consequently, increasing vaccination uptake among adolescents in HK. The Health Belief Model (HBM), which has been used in previous HPV vaccination–promotion interventions, was employed as the underlying framework to develop the programme [9]. The HBM consists of six core concepts, namely perceived susceptibility, perceived severity, perceived benefits, perceived barriers, cues to action, and self-efficacy, all of which were used to guide programme development [10]. Moreover, experts from a variety of disciplines including nursing, paediatrics, pharmacy, and education were involved in content curation. The programme will be delivered at secondary schools in the form of education sessions comprising varied components including informative animations, interview videos, small group discussions, a booklet, and an online game. Topics covered will include an introduction to HPV infection, transmission methods, diseases caused, incidence of cervical cancer, prevention measures, efficacy and protection provided by HPV vaccination, common misconceptions and concerns, and information on vaccine pricing, location, and availability. As user-design methods were adopted in the development of the programme, the animations, interview videos and booklet produced by the research team were distributed to potential participants prior to programme commencement, and interviews were conducted to collect their views on resource acceptability and effectiveness. The current study aims to describe participant feedback, highlight the benefits and limitations of the resources and ultimately, provide guidance for the development of educational programmes in future health promotion initiatives.

## Methods

### Design

A qualitative exploratory approach was adopted to conduct an in-depth investigation of the attitudes of adolescents, family and school staff [11].

### Objective

To assess user feedback on the quality, acceptability and effectiveness of MDL-SHPVP educational resources.

### Participants

A purposive sampling strategy was used to recruit participants in order to accurately represent the views of potential users and facilitators of the MDL-SHPVP. As HPV vaccination is more urgent for females due to the high risk of developing cervical cancer and because prior evidence suggests that mothers are the main decision-maker in accepting and initiating HPV vaccination [12], the primary target participants for this study were adolescent daughters and their mothers. As data triangulation was carried out to increase finding credibility, school staff who could facilitate programme implementation or influence the uptake of HPV vaccination were also invited to join the study, and at least one person was recruited from each group. A total of 26 participants were successfully recruited, including eight mother-daughter dyads, four teachers, three social workers, two school principals and one school nurse. Recruitment for adolescents targeted females enrolled in a local secondary school who were aged 14–17 years (Grades 8–11) and communicable in Chinese. Adults were recruited based on their relation to the student and their identity as potential facilitators of the educational programme.

### Programme Development

The MDL-SHPVP was formulated with the aim to increase participants’ understanding of HPV and HPV vaccination and promote vaccine uptake among adolescents. The programme primarily targets adolescents and parents and also involves school staff such as teachers, school nurses and principals. According to the literature, a lack of information on HPV and HPV vaccination is frequently linked with non-vaccination, acting as a significant obstacle in vaccine uptake [13]. To address this barrier, a variety of informative resources which could raise participant confidence regarding HPV vaccination and facilitate their decision-making process were developed.

In total, six interview videos with health care professionals (HCPs), three animations, four sharing videos by vaccinated students and their parents, an information booklet and a digital game were developed after conducting an extensive literature review of academic articles and local news publications which provided evidence on current attitudes, barriers and facilitators of HPV vaccination. Paediatric health and education experts were also consulted and involved in resource development. A summary of the programme resources is depicted in Table 1.

**Table 1:** Summary of educational resources developed for the MDL-SHPVP

Resource type	HCP interview videos	Animations	Sharing videos of vaccinated students and parents	Booklet	Digital game (Kahoot)
Topic	•Mechanism of HPV infection and HPV vaccination, addressing vaccine safety and efficacy concerns	•Common attitudes, beliefs, myths and misconceptions related to HPV and HPV vaccine •Cervical cancer and its stages •Process of receiving HPV vaccination	•Experience of receiving HPV vaccination and decision-making	•Summary of animations and video content •Guide to arranging HPV vaccination	•Knowledge of HPV and HPV vaccination
Content description	•6 videos (total duration approx. 80 min) •Interviews with 3 nurses, 2 paediatricians and 1 pharmacist •HCPs share their knowledge of HPV infection, the associated diseases, their views on common concerns about HPV vaccines, and their own experience of receiving HPV vaccination	•3 animations (total duration approx. 11 min) •Discussions about HPV infection and the vaccination between cartoon characters including adolescents, their parents, a school nurse and a doctor •Set in various scenes relevant to adolescents including a school, clinic and park	•4 videos (total duration approx. 18 min) •Individual sharing by parents and students about their decision to get the HPV vaccine for themselves or their daughters and their vaccination experiences	•Covers information about HPV infection, cervical cancer, HPV vaccines and provides details on arranging vaccination in Hong Kong •Includes Q&A section	•Aimed at testing and reinforcing the knowledge adolescents gained from the animations, videos, and booklet

*HCP* health care professional, *HPV* human papillomavirus.

### Data Collection

Intervention materials which included six HCP interview videos, three animations, three sharing videos and a booklet were distributed to participants for their evaluation. After viewing the resources, individual semi-structured interviews were carried out over the telephone by a trained research assistant from January to March 2021 to collect participant feedback. Interviews were conducted over telephone instead of face-to-face due to social distancing requirements arising from the COVID-19 pandemic. Interviews took place in Cantonese and ranged from 15 to 48 min. All interviews were audio-recorded with consent. An interview guide was developed and questions were designed to explore whether the resources met participants’ personal learning needs, to identify potential improvements in knowledge of HPV and HPV vaccination, changes in attitudes and intentions toward HPV vaccination, views of other programme components such as small group discussions and suggestions for improving the resources. Regarding the evaluation of knowledge, the participants were asked to describe the main message they received from each resource. If they were able to describe the messages accurately (descriptions matched with the information provided in the resources), the resources were considered to be effective in raising participant knowledge levels. Examples of interview questions include: “Could you explain how the MDL-SHPVP resources were able to enhance your knowledge and clear up misconceptions about HPV infection and HPV vaccination?”, “If you have the option to promote HPV-related information and HPV vaccination to other students and parents, which components of the MDL-SHPVP resources and key concepts will you use and why?”, and “What is one thing that you would change regarding the MDL-SHPVP resources?”. Data collection was stopped once the point of thematic saturation was identified during data analysis.

### Data Analysis

Interviews were transcribed verbatim and translated into English by a bilingual research team member. Translations were checked and verified by bilingual research team members to ensure accuracy in meaning and message. The six-phase framework devised by Braun and Clarke (2006) was applied for thematic analysis [14]. Transcripts were coded inductively line by line, then grouped under main themes and sub-themes by a research assistant. Further review of the transcripts and resulting themes was carried out by remaining research team members to establish credibility of findings. Data collection was concluded once thematic saturation was reached, defined as the point where no new themes were identified from the interviews.

## Results

A total of 26 potential users and facilitators of the MDL-SHPVP were interviewed. Three main themes and six sub-themes emerged from the data as summarized in Table 2.

**Table 2:** Themes and sub-themes emerging from participant feedback on programme resources

Themes	Sub-themes
Strengths	Informative and comprehensive content Positive impact on attitude towards HPV vaccination
Concerns	Doubts about effectiveness of proposed small group discussion component
Limitations	Remaining doubts and perceived ambiguity of content Accessibility barriers Implementation challenges

*HPV* human papillomavirus.

### Strengths

#### Informative and Comprehensive Content

Prior to the study, most participants had little to no knowledge about HPV and HPV vaccination. Some had heard about them vaguely from television advertisements but only possessed superficial knowledge.

I never really paid attention to this topic so I don’t know what the HPV vaccine is for. (Student 2)

I’d always see promotions for the vaccine but didn’t know how long the protection would last. I also heard about really horrible kinds of negative side effects so it felt like the risks involved with the vaccine were very high. (Social worker 2)

After viewing the programme resources, they felt that they were able to gain extensive information about the virus and the vaccination, as well as clear up any misconceptions.

These resources are definitely helpful. Although we have HPV vaccine promotions on TV, I don’t know if young people nowadays actually watch those. So, if they are provided with such comprehensive materials, it can answer a lot of questions that people might have, misconceptions can be cleared, and parents can be more informed. (Teacher 3)

A lot of people mix up concepts of prevention and cure so I felt these were explained clearly in the resources. (Student 2)

Specifically, the participants noted that they learnt about the difference between the 2-, 4-, and 9-valent vaccines, their efficacy rates, the ideal age for vaccination, diseases caused by HPV infection, low occurrence of negative side effects, applicability to both women and men and more. One participant also noted that after watching the videos, they had more confidence in their ability to explain the topic to others while some suggested that it was able to illustrate the personal relevance of the vaccination.

If you see a commercial [about HPV] you would just watch and think it’s unrelated to you and just move on, but after seeing these materials you would know more and perceive it as a more personal problem and give it more thought. (Teacher 1)

I think [the resources] are quite useful especially for parents since they are the ones making the vaccination decision… it can help them understand the situation more and let them evaluate their options. (School nurse)

The booklet on HPV, however, was the most popular resource, as it was considered to be the most comprehensive and convenient source of information. The participants often preferred the booklet over the videos as they felt it already contained information mentioned in the videos, could be viewed at their own pace and was not as time-consuming as the videos.

I liked the booklet because it summarized everything and if people miss any points from the videos, they can catch up on those easily. (Student 2)

I think the booklet is a must as people can take it home and digest it slowly. (Teacher 5)

Among the videos, views towards the animations and HCP interviews were also favourable. The animations were thought to be suitable and engaging for students while the HCP interviews were more attractive to parents.

The videos might be better than the booklet because sometimes the things we distribute do not necessarily end up in parents’ hands. (Teacher 3)

I prefer the animations because the length was appropriate and the information was relevant as it mentioned the usefulness of the vaccination. (Student 7)

The sharing videos with the vaccinated students and parents, however, were poorly received. Most felt that the videos did not add any further information and that if they wanted to hear about vaccination experiences, they could simply inquire about it from their own friends or family.

The sharing videos weren’t really helpful because I could just ask those around me and probably get the same opinions. (Student 5)

#### Positive Impact on Attitude Towards HPV Vaccination

The resources had a favourable effect on the participants’ attitudes towards HPV vaccination. They suggested that the content was able to increase their confidence in the safety of the vaccines and improve vaccination intention. The boost in confidence was frequently attributed to the content in the HCP interviews, as the advice of health experts was considered trustworthy and persuasive.

I’m open to vaccination because the HCPs in the videos also consider it safe. (Teacher 1)

The HCP interviews mentioned that the vaccines have undergone various studies and are already being used in other countries so I feel more confident about them. (Student 4)

Moreover, the importance and personal relevance of early vaccination was also successfully conveyed as several participants expressed a newfound realization of the urgency of vaccination.

Since I’m now 14 years old, it would be good for me to get vaccinated soon since it’s recommended for 9-14 years old and the immunity generated would be higher. It makes me want to get vaccinated. (Student 3)

[The resources] made me feel like I must get vaccinated…I think it’s worth it for adolescents to get vaccinated as soon as possible as it will have higher effectiveness. (Social worker 2)

One parent, however, noted that although they were convinced of the necessity of the vaccination, they remained unsure regarding the urgency.

The materials didn’t convince me that the vaccination needs to be done sooner. Didn’t feel like I need to arrange it immediately. (Parent 4)

### Concerns

#### Doubts About Effectiveness of Proposed Small Group Discussion Component

Besides the videos and booklet, the participants were also asked to comment on the feasibility of small group discussions including parents, students and health care workers at schools. Responses to the suggestion were largely lukewarm. Although most believed that interactions between the participants could encourage greater exchange and retention of information, there were frequent concerns regarding parent availability and students’ willingness to participate actively.

[Group discussions] might be a bit difficult as you need people to proactively ask questions, answer and talk to each other, so you might need to assess whether or not people are enthusiastic about the topic and willing to communicate. (Student 2)

Besides issues of participation, it was also suggested that the participants may find it difficult to ask suitable questions due to their lack of background knowledge on the issue and felt that more questions may emerge after the intervention.

If [participants] don’t know much about HPV, they won’t really know which questions to ask. You need to have some sort of basic awareness in order to come up with follow-up questions… but also, if they are given some information right before the discussion, I don’t know if they will have enough time to digest it. (Teacher 5)

However, some participants believed that group discussions may be effective as the participants could directly discuss their concerns with HCPs and possibly encourage each other to get vaccinated.

I think it’s a good method since parents and students can directly ask questions from the HCP instead of finding incorrect answers from their own research. (Student 4)

“[Group discussions] can be effective as they are direct, face-to-face, and you could get to know information first-hand. It would be good as long as they have one part where they give out information and one part for Q&A.” (Parent 6)

### Limitations

#### Remaining Doubts and Perceived Ambiguity Of Content

Despite overall satisfaction with the resources, some doubts about the vaccination still remained among participants. The most common concerned the 10-year protection duration of existing vaccines. Though it was explained that the current estimation of the duration is based on available research which only extends to about 10 years, some adults still appeared sceptical of the value of early vaccination as they were worried that adolescents would not be protected after the 10-year period.

I saw that the protection duration lasts ten years so I’m unsure about what time is actually best for vaccination. (Social worker 2)

They said the protection duration is at least 10 years, but if my daughter is 14 or 15 and already vaccinated, then what will she do when she is 25? Is [the vaccination] meaningless then? Will she need to get a booster dose? (Parent 7)

In addition, the participants also raised the issue of whether the vaccination was genuinely required for boys and whether booster shots were necessary after getting low-valence vaccines in order to provide protection against more HPV subtypes.

The resources say that boys can also be infected but don’t talk about the diseases that they could get… so this doesn’t clear up whether or not boys actually need the vaccination. (Parent 8)

For people who’ve already had the low-valence vaccines, I wanted to ask if a booster shot with the 9-valent vaccine is needed? (Social worker 3)

#### Accessibility Barriers

The most common barrier encountered by the participants was a lack of subtitles which they felt hindered their ability to fully grasp video contents, as they might not be able to hear all the points, or miss out due to natural lapses in concentration.

If people aren’t focusing, they might miss key points. If there are subtitles and we can see what they’re talking about, it’ll be easier to remember. (Parent 6)

Since the videos didn’t have subtitles, I wasn’t really able to get the message unless I really paid attention. (Social worker 3)

In fact, a few responses indicated that some participants indeed failed to grasp the key messages from the resources. For instance, although potential negative side effects were described in the videos and mentioned in the booklet, this aspect seemed to have been overlooked by a teacher.

Did the videos talk about any negative side effects? (Teacher 3)

#### Implementation Challenges

Challenges in intervention delivery, particularly in the context of the COVID-19 pandemic, were also identified. As school suspensions have been frequent and classes are often shifted online, some participants suggested that the programme could be delivered through online communication tools. Particularly for parents, it was believed that the convenience of online meetings may lead to increased participation.

Since we have started having meetings online during the pandemic, I think it might be easier to recruit people to join online sessions. (School nurse)

I’m not sure if parents would be willing to attend in person… maybe they’d be more willing to attend on Zoom. If it’s just a presentation and a Q&A section, the response may be better if it’s arranged online. (Parent 1)

In contrast, two teachers felt that online meetings would not be entirely effective, as there was a risk of non-attendance, and that interpersonal communication would be significantly affected, therefore reducing the interactive nature of the intervention.

Though it might be a bit easier to contact parents through Zoom these days, it’s still difficult to get them registered [to attend]. (Teacher 3)

“The thing is that the average parent will generally have no idea about this topic and using online communication tools is a bit passive…” (Teacher 5)

## Discussion

As part of a larger randomized controlled trial to evaluate the effectiveness of the MDL-SHPVP in raising HPV vaccination rates among female adolescents in Hong Kong, tailored educational resources were developed following the identification of relevant participant learning needs [7]. Resources primarily aimed to clear up misconceptions and provide essential information about HPV infection and HPV vaccination to potential vaccine recipients and facilitators. The acceptability and effectiveness of the resources in raising knowledge levels and improving attitudes towards HPV and HPV vaccination were assessed in the current study. Findings suggest that the resources were well-received and that the educational programme could be effective in improving participants’ knowledge of HPV, generating positive attitudes towards HPV vaccination, and consequently, boosting vaccination intention. These are promising results which may hint at potentially increased vaccination uptake upon programme implementation in Hong Kong.

In terms of the educational value of the resources, the participants believed that their learning needs were met successfully and that they received vital and relevant information on the topic. It was noted that the resources were comprehensive and allowed them to clarify their understanding of certain key concepts such as the differences between strategies of prevention and cure. This is a positive sign, as several studies on predictors of HPV vaccination uptake suggest that heightened knowledge of the virus and vaccination are positively correlated with vaccination intention [15, 16]. Moreover, as further evidence of the benefits of the resources, a participant expressed that they now felt confident in explaining the topic to others, which was an encouraging indicator of the resources’ ability to not only fulfil learning needs, but also to generate vital conversations about health amongst the community. As previous studies emphasize the role of communication in facilitating HPV vaccination and demonstrate that peer-to-peer and parent-to-parent conversations are valuable in promoting vaccine uptake [17], our results suggest that the resources could empower participants to promote the vaccine on their own initiative and positively influence vaccination rates.

Additionally, the participants also reported improvements in their attitude towards HPV vaccination. While previous findings show that a fear of negative side effects among parents and adolescents acts as a major deterrent in initiating HPV vaccination [18, 19], the programme resources were able to effectively address participants’ concerns as they expressed heightened trust in vaccine safety and demonstrated stronger vaccination intention. However, though progress was also made regarding participants’ perception of the necessity of early vaccination, one parent expressed that they did not feel the urgency to get their daughter vaccinated and felt it would be better to wait, reflecting a “wait-and-see” attitude towards vaccines which has previously also been reported among Hong Kong parents [20]. It is suggested that more effective communication to back up the recommendation of early vaccination is needed to persuade parents to initiate vaccination for their children sooner.

Besides the content of the resources, the diverse types of presentation were also well-accepted by the participants. The animations were found to be engaging and appropriate for adolescents, while the HCP interview video was more popular with the parents. The involvement of HCPs was especially compelling as the participants considered them to be authoritative sources of health information and trusted their knowledge on HPV vaccination. This is reflective of findings from past studies which have emphasized the central role played by health providers in vaccination promotion efforts [21, 22]. Moreover, besides the animations and HCP video, the booklet was also a favoured resource as the participants felt it was a convenient point of reference that summarized content from the videos and included a question and answer section which could address their queries in a concise manner.

Some limitations with the programme resources and proposed intervention components were noted, however. Regarding the videos, the participants felt that the content in the parent and student sharing videos was repetitive, and that the lack of subtitles hindered comprehension. To address these issues, subtitles will be added to the videos to enhance legibility and facilitate audience understanding, and the sharing videos will be used selectively according to participant needs. As there was also uncertainty regarding the effectiveness of the small group discussion component, process evaluation will be carried out after programme implementation to identify strengths and potential areas of improvement. However, with the requirement of social distancing during COVID-19 outbreaks, teachers voiced concerns about the possibility of low engagement online, suggesting that though online communication may facilitate contact with parents, there was no guarantee of attendance and overall interactivity may also be reduced. Additionally, some participants remained sceptical of the information provided, including uncertainty about the duration of protection offered by the vaccine, and whether it was necessary for boys to also receive the vaccination.

## Limitations

This study has limitations. As a purposive sample was recruited for this study, the representative nature of the sample may be affected. Since interviews were conducted over the telephone instead of face-to-face, the possible presence of mothers during their daughters’ interviews may affect response quality. Moreover, the investigation could have benefited from the supplemental use of quantitative data collection, particularly in identifying and comparing participants’ views towards each separate educational resource.

## Conclusion

The participants demonstrated high acceptability of the MDL-SHPVP resources. They appreciated the quality and content of the programme resources and felt that they were effective in raising knowledge levels and promoting HPV vaccination. Recommendations provided to further improve the materials have been implemented accordingly. Study findings may aid the development of other education-based health interventions targeted at adolescents and generate a positive impact on youth health in society.

## Declarations

Study approval was granted by the relevant university ethics committee (Ref. no: 2019.55). The rights and safety of participants were protected in accordance with local laws, the Declaration of Helsinki, institutional policies and the ICH-GCP. Written consent was obtained from the participants, and they were informed of their right to refuse participation or withdraw from the study at any time. All the data were encrypted and stored confidentially with access provided only to research team members.
